# Intake and sources of dietary fatty acids in Europe: Are current population intakes of fats aligned with dietary recommendations?

**DOI:** 10.1002/ejlt.201400513

**Published:** 2015-08-19

**Authors:** Ans Eilander, Rajwinder K. Harika, Peter L. Zock

**Affiliations:** ^1^ Unilever Research and DevelopmentVlaardingenThe Netherlands; ^2^ Top Institute Food and NutritionWageningenThe Netherlands

**Keywords:** Adults, Dietary fat, Europe, PUFA, SFA

## Abstract

The development of food‐based dietary guidelines for prevention of cardiovascular diseases requires knowledge of the contribution of common foods to SFA and PUFA intake. We systematically reviewed available data from European countries on population intakes and dietary sources of total fat, SFA, and PUFA. Data from national dietary surveys or population studies published >1995 were searched through Medline, Web of Science, and websites of national public health institutes. Mean population intakes were compared with FAO/WHO dietary recommendations, and contributions of major food groups to overall intakes of fat and fatty acids were calculated. Fatty acid intake data from 24 European countries were included. Reported mean intakes ranged from 28.5 to 46.2% of total energy (%E) for total fat, from 8.9 to 15.5%E for SFA, from 3.9 to 11.3%E for PUFA. The mean intakes met the recommendation for total fat (20–35%E) in 15 countries, and for SFA (<10%E) in two countries, and for PUFA (6–11%E) in 15 of the 24 countries. The main three dietary sources of total fat and SFA were dairy, added fats and oils, and meat and meat products. The majority of PUFA in the diet was provided by added fats and oils, followed by cereals and cereal products, and meat and meat products.

**Practical applications:** While many European countries meet the recommended intake levels for total fat and PUFA, a large majority of European population exceeds the widely recommended maximum 10%E for SFA. In particular animal based products, such as dairy, animal fats, and fatty meat contribute to SFA intake. Adhering to food‐based dietary guidelines for prevention of CHD and other chronic diseases in Europe, including eating less fatty meats, low‐fat instead of full‐fat dairy, and more vegetable fats and oils will help to reduce SFA intake and at the same time increase PUFA intake.

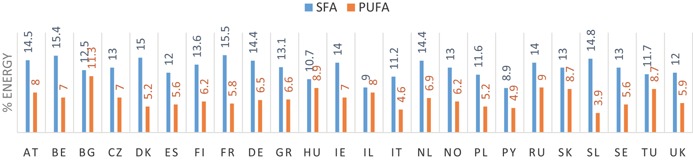

In European countries, SFA intakes are generally higher than the recommended <10%E and PUFA intakes lower than the recommended 6–11%E. Adhering to food‐based dietary guidelines for prevention of CHD and other chronic diseases including eating leaner variants of meat and dairy, and more vegetable fats and oils will help to decrease SFA intake and increase PUFA intake.

**Abbreviations**
ALA: α‐linolenic acid
CHD: coronary heart disease
DHA: docosahexaenoic acid
EPA: eicosapentaenoic acid
FAO: food and agricultural organization
LA: linoleic acid
PUFA: polyunsaturated fatty acids
SFA: saturated fatty acids
TFA: trans‐fatty acids
WHO: World Health Organization

## 1 Introduction

Most dietary recommendations to prevent chronic diseases such as coronary heart disease (CHD) focus on the reduction of saturated fatty acid (SFA) intake. Animal foods are quantitatively the most important dietary sources of SFA intake 1. Dietary recommendations by FAO/WHO 2 as well as the European Society of Cardiology 3 and European Commission Eurodiet Core report 4 advise an upper limit of 10% of energy intake (%E) for total SFA intake and an intake range of 6–11%E for PUFA. In practice, dietary PUFA are mainly linoleic acid (LA) and some α‐linolenic acid (ALA) from vegetable oils. The long chain PUFA eicosapentaenoic acid (EPA) and docosahexaenoic acid (DHA) from marine sources contribute very little to total PUFA intake, a few hundred milligrams as compared with up to dozens of grams for LA plus ALA.

Guidelines for total fat are quite liberal, FAO/WHO recommends a range of 20–35%E 2 and some others up to 40%E (or no upper limit at all) because there is no strong evidence that, unlike dietary fatty acid composition, the total amount dietary fat is related to CVD or other chronic diseases 5.

There are several reports on intakes of fat and fatty acids in European countries and other populations 1, 6, 7, 8, 9. The most recent reviews 7, 9 concluded that in the majority of countries, the reported average SFA intake was higher than the recommended maximum of 10%E, while in half of the countries average PUFA intake was lower than the recommended range of 6–11%E. However, the recent reviews 8, 9 do not report on the main dietary sources of SFA and PUFA across countries. Data on the main dietary sources of SFA and PUFA are needed to help authorities translating recommendations on nutrients into practical food‐based dietary guidelines for the general public.

By means of a systematic review we collected population representative data for different European countries on total fat, SFA, and PUFA intakes, and their dietary sources. We compare reported intake levels of SFA and PUFA with the amounts on fat and fatty acid intake as recommended by the FAO/WHO. We also report information on the main dietary sources of fats and fatty acids in Europe.

## 2 Methods

### 2.1 Search strategy

To retrieve information on dietary fat and fatty acid intakes and sources in adults, we used the European surveys from the publication by Harika et al. 9 as basis. A literature search was performed in Medline and Web of Science (January 2012–March 2015) to identify the latest publications, following a search string with the terms: (“total fat,” “saturated fat*,” or SAFA, “polyunsaturated fat*” or PUFA, “monounsaturated fat*” or MUFA) and (consumption or intake or survey) and (adult* or population). Reference lists of all articles of interest were checked for additional studies. No language restrictions were used. In addition to the search via Medline and Web of Science, national intake data were searched through websites of national public health institutes (see for more details Harika et al. 9).

After the search, all the publications and reports were screened to determine eligibility of data based on the following inclusion criteria: (i) Data applied to a country within European region as defined by WHO, (ii) national surveys or population‐based observational studies with a sample size of ≥100 measuring dietary fatty acid intakes, (iii) published later than 1995, (iv) data from general adults population (>18–85 years), and (v) complete information provided on intake of total fat and SFA, MUFA, and PUFA. For countries where multiple datasets were available, data from the most recent national dietary survey were included. If national dietary surveys were not available, representative data from population‐based observational studies were used.

### 2.2 Dietary recommendations for intakes of fatty acids

The mean population intakes per country were compared to the recommended intake levels and ranges for total fat and fatty acids for adults as determined by the FAO/WHO 2 for total fat 20–35%E; for SFA <10%E, and for PUFA 6–11%E.

### 2.3 Data extraction and statistical analysis

From each source of intake data per country, we extracted the means and when reported also standard deviations (SD) of intakes of total fat, SFA, MUFA, and PUFA. Where fat and fatty acid intakes were expressed as absolute amounts (grams per day), values were converted to percentage of total energy intake using the conversion factor of 37.7 kJ/g for fat and fatty acids.

Where data were reported for subgroups (e.g., by age range or by gender), a weighted mean for adults was calculated by weighing the mean intake of each subgroup by the number of the subjects in the subgroup. When SDs were not reported, they were calculated using the population sample size and reported standard error of mean (SEM).

### 2.4 Dietary sources of total fat, SFA, and PUFA

To identify and compare the main dietary sources of total fat, SFA, and PUFA in different European countries, we defined four food groups that were expected to significantly contribute to the intake of total fat, SFA, and PUFA. These included: (i) all milk and milk products including yoghurt, cream, and cheese, clustered as “dairy;” (ii) all meat and meat products, including meat dishes, processed meats, and poultry, clustered as “meat and meat products;” (iii) oils, margarine, butter, spreads, other fats, dressings, sauces, and mayonnaise, clustered as “added fats and oils;” and (iv) breads, cereals, rice, pasta, grains, clustered as “cereals and cereal products.” We recorded, but did not specify the remaining food groups contributing to the intake of total fat, SFA, and PUFA because these contributed much less to intake and varied considerably between countries. In addition, the remaining food groups were heterogeneously defined between countries, which made it difficult to make comparisons.

## 3 Results

### 3.1 Availability of data

Our earlier review by Harika et al. 9 provided 17 publications with population intake data from Europe. The additional literature search yielded 16 publications and reports that met the inclusion criteria for potentially eligible data sources. Of these 16 data sources, 8 were excluded because they concerned duplicate data (*n *= 5) or did not contain complete information on fatty acids (*n *= 3). Thus, a total of 25 publications were included in the current review. Each data source represents one country, except Poland for which we used two data sources, one for intake data 10 and one for dietary sources 11.

These publications reported population intake data on total fat and fatty acid intake from 24 European countries (Table 1). For 11 of these 24 countries, also dietary sources of total fat and SFA intake were reported, and for 10 also dietary sources of PUFA. For 16 countries data were derived from national surveys, for the 8 other countries data from large studies with representative population samples were used.

**Table 1:** Data sources and mean percentage of total energy intake (%E) and standard deviation (SD) of intakes of total fat, SFA, MUFA, and PUFA among adults in 24 European countries

Country	Year	Data source	Sample size	Dietary method	Age (years)	Energy Kcal	SD	Total fat (%E)	SD	SFA (%E)	SD	MUFA (%E)	SD	PUFA (%E)	SD
Austria (AT) 26	2009	Austrian Nutrition Survey 2008	2123	24 h recall	19–64	1970	n/a	37	n/a	14.5	n/a	12.5	n/a	8	n/a
Belgium (BE) 27	2010	Belgian National Food Consumption Survey (BNFCS)	873	2 × 24 h recall and FFQ	19–59	2016	679	37.6	5.9	15.4	2.9	13.7	2.0	7	2.3
Bulgaria (BG) 18	1998	National Nutrition Survey	860	24 h recall	18–60	2379	903	34.6	8.6	12.5	5.5	9.9	3.2	11.3	4.6
Czech Republic (CZ) 28	2010	The HAPIEE Study	7913	FFQ	45–69	2018	716	36	15.1	13	5.7	13	5.5	7	2.9
Denmark (DK) 29	2010	National Dietary Survey	3151	7 days food record	18–75	2173	668	35	5.6	15	3	12	2.4	5.2	1
Finland (FI) 30	2012	National Nutrition Survey (FINDIET 2012)	1708	48 h dietary recall data	25–64			35.4	8	13.6	4.1	12.6	3.65	6.2	2.4
France (FR) 31	2006–2007	Etude Individuelle Nationale des Consommations Alimentaire (INCA 2)	1918	7 days dietary record	18–79	2066	558	39.1	5.7	15.5	5.5	13.9	4.4	5.8	2.4
Germany (DE) 32	2004	German Nutrition Survey	>1000	Written diet record	19–65	2460	n/a	35.9	n/a	14.4	n/a	12.8	n/a	6.5	n/a
Greece (GR) 33	2004	Greek EPIC Cohort	20 942	FFQ	25–64	2245	656	46.2	5.3	13.1	2.7	22.3	4	6.6	2.6
Hungary (HU) 34	2011	National Dietary Survey	3077	3 days dietary record	31–60	2455	648	37.5	5.5	10.7	2.4	11.3	2.7	8.9	2.2
Ireland (IE) 35	2008	Food Consumption Survey	1097	7 days food dairy	18–65	n/a	n/a	35.8	5.4	14	3.1	12	2	7	2.1
Israel (IL) 36	2001	MABAT First Israeli National Health and Nutrition Survey	3242	24 h recall and questionnaire	25–64	1856	882	33	9	9	4	11	5	8	4
Italy (IT) 37	2011	INRAN SCAI National Food Consumption Survey	1313	3 days dietary record	19–64			36.4	5.3	11.2	2.4	17.5	2.4	4.6	3.3
Netherlands (NL) 38	2011	Dutch National Food Consumption Survey	2106	24 h recalls	19–65		n/a	36.7	n/a	14.4	n/a	13	n/a	6.9	n/a
Norway (NO) 39	2012	Norkost 3	1787	24 h recalls and FFQ	18–70	2245	n/a	34	7	13	3	12	3	6.2	2.3
Poland (PL) 10	2003	Household Food Consumption Survey	2893	24 h recall	19–65	n/a	n/a	35.7	8.1	11.6	4.1	15.4	4.5	5.2	2.4
Portugal (PT) 40	1999	Cross‐Sectional Study	489	FFQ	>40	2316	668	28.5	5	8.9	2.4	12.4	2.4	4.9	1.1
Russia (RU) 28	2010	The HAPIEE Study	9098	FFQ	45–79	2579	763	43	15.5	14	6.2	16	6.1	9	3.5
Slovakia (SK) 41	2002	Two Epidemiologic Studies	4018	24 h recall	25–60	2185	n/a	38.5	n/a	12	n/a	15.9	n/a	5.6	n/a
Slovenia (SI) 42	1999	Epidemiological Study	2183	FFQ	19–80	n/a	n/a	35.5	n/a	13	n/a	11.9	n/a	8.7	n/a
Spain (ES) 33	2005	Large Studies (pooled analysis of studies) 1990–1998	10 208	24 h diet, 3 days diet record and FFQ	18–65	2727	n/a	44.3	n/a	14.8	n/a	13	n/a	3.9	n/a
Sweden (SE) 43	2012	Riksmaten; Adults Dietary Survey	1797	4 days food dairy	18–80	1987	623	34.2	6.4	13	3.2	12.8	2.8	5.6	1.9
Turkey (TU) 44	2014	Nutrition and Health Survey	8058	24 h recall	>19	1909	792	34.4	9.9	11.7	6.9	12.2	6.9	8.7	5.9
United Kingdom 45	2012	National Diet and Nutrition Survey	434	4 days estimated food diary	19–64	1803	558	32.9	7	12	3.4	11.7	3	5.9	2

### 3.2 Reported intakes of total fat and fatty acids and differences with recommended intake levels

#### 3.2.1 Total fat

Mean intake of total fat ranged across countries from 28.5 to 46.2%E (Table 1), with reported intakes being the lowest in Portugal and the highest in Greece. In 9 of the 24 countries, the mean total fat intakes were within the recommended range of 20–35%E, whereas it was higher than 35%E in the other 15 countries.

The main dietary sources of total fat were added fats and oils (which contributed 9–46% to total fat intake across countries), meat and meat products (17–26%), and dairy (11–24%). In the UK, Finland, and Netherlands, also cereals and cereal products contributed substantially to total fat intake (10–18%). Also, cakes, pastries, and desserts were often reported as contributors to total fat intake (data not shown).

#### 3.2.2 SFA

Mean intakes of SFA ranged from 8.9 to 15.5%E across countries, with the lowest intake reported in Portugal and the highest intake in France (Table 1). In two of the 24 countries (Portugal and Israel), the mean SFA intakes were below the FAO/WHO recommended intake of less than 10%E.

**Figure 1:**
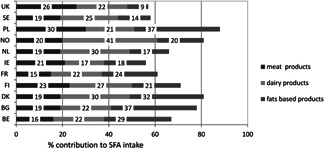
Main food groups contributing to total reported SFA intake in 11 European countries. *BG: mean of the range is used. BE, Belgium; BG, Bulgaria; DK, Denmark; FI, Finland; FR, France; IE, Ireland; NL, Netherlands; NO, Norway; PL, Poland; SE, Sweden; UK‐United Kingdom.

The main dietary sources of SFA were dairy (contributing 17–41% to total SFA intake), fats and oils (9–37%), and meat and meat products (15–30%) (Fig. 1). In the UK and Finland, cereals and cereal products contributed 16–18% to total SFA intake (data not shown). Cake and pastry/desserts and sugar/preserve confectionary were the main remaining food groups contributing to SFA intake (data not shown).

#### 3.2.3 PUFA

Mean intakes of PUFA ranged from 3.9 to 11.3%E, with the lowest intake in Spain and the highest intake in Bulgaria (Table 1). In 14 of 24 European countries, PUFA intakes were within the FAO/WHO recommended range of 6–11%E.

**Figure 2:**
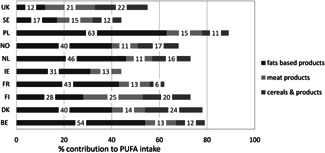
Main food groups contributing to total PUFA (LA + ALA) intake in ten European countries BE, Belgium; DK, Denmark; FI, Finland; FR, France; IE, Ireland; NL, Netherlands; NO, Norway; PL, Poland; SE, Sweden; UK‐United Kingdom.

The main dietary sources of PUFA were added fats and oils (contributing 12–63% to total PUFA intake), meat and meat products (11–25%), and cereals and cereal products (6–24%) (Fig. 2). In the UK and Ireland, also potato chips contributed substantially to PUFA intake (11–18%) (data not shown). Similar to total fat and SFA, the remaining dietary sources contributing to PUFA intake varied markedly between countries. The most important remaining food groups that contributed to PUFA intake were cakes/pastry and vegetables and vegetable dishes (data not shown).

## 4 Discussion

Our review shows that the average population intakes of SFA and PUFA does not meet recommended intakes Europe. In only 2 of the 24 European countries included in this review SFA intake was below the recommended maximum intake of 10%E. PUFA intakes were below the optimal intake range in about half of these countries. The main dietary sources that contribute to total fat and SFA intake in Europe are dairy, added fats and oils (including animal fats such as butter, lard, beef drip), and meat and meat products. The main contributing dietary sources to intake of PUFA are added fats and oils (including vegetables oils, margarines, and mayonnaises), meat and meat products, and cereals and cereal products.

Only few publications have evaluated data on average fat and fatty acid intakes in populations from different countries 1, 8, 9, 12. Also, recent reviews on this topic 8, 9 did not the address foods that deliver total fat and fatty acids to the diet. The current review used a systematic approach to summarize available data on intakes of SFA and PUFA and their dietary sources in Europe. Several limitations of our analysis should be considered when interpreting our findings, both for estimated intakes within individual countries and for making comparisons between countries. The main limitation is that the available data sources are heterogeneous with respect to the sampling and dietary assessment methods. National dietary surveys are the preferred type of research to estimate the distribution of nutrient intake in populations. These were available for the majority (16 out of 24) of the countries. However, the methodology of national dietary surveys differ between countries and each has its specific limitations. Data from large observational studies were used for eight countries without data from a national dietary survey. For half of the countries in this review, data were based on 3–7 day weighed food records, multiple 24 h dietary recalls, and 24 h dietary recalls that were combined with food frequency questionnaires (FFQ). This combination of dietary assessment methods is considered more reliable for estimating the usual intake of foods in individuals than only single FFQs or 24 h recalls 13, 14, 15, which were the basis for data from the other half of the countries. For Poland the data on dietary sources were based on the Household Budget Survey, which does not take into account food that is lost in the household from purchase to actual consumption 11. The heterogeneity in design and size of the studies, may explain part of the large variation in total fat and SFA intake between the neighboring countries Spain and Portugal. It should be noted that data for Portugal were derived from a much smaller study than for Spain.

Another important limitation of our review is incomplete information on specific fatty acids for some foods in local food composition tables 16. This can easily lead to underestimation of true intakes of SFA, MUFA, or PUFA, which is probably also the case for the current data, because the sum of SFA, MUFA, and PUFA was in all countries lower than that of total fat (Table 1). Such differences are also found in other studies 1, 8, 9, 12. For four countries in our review this difference was ≥5%E (Israel 5%E, Spain 5%E, Hungary 6.6%E, and Slovenia 12.6%E). A larger difference between total fat and the sum of SFA, MUFA, and PUFA could indicate that a higher number of foods in the food composition table are lacking values for fatty acids. Therefore, results for classes of fatty acids are less reliable than for total fat.

A third limitation is that definitions of food groups are not consistent across countries, which may affect the estimated contribution of the different food sources to fat and fatty acids intake. For example in France, Netherlands, Sweden, and Ireland, we grouped the condiments and sauces (mainly mayonnaise) with “added fats and oils,” whereas for other countries without a specific “condiments and sauces” food group, information was lacking on whether these were grouped with added fats and oils or not. The fact that food groups were not consistently defined between countries was the reason that we found that in the UK and Finland, also cereal and cereal products contributed significantly to SFA intake next to added fats and oils, dairy, and meat products. In these two countries, this food group also included cakes, pastries, biscuits, pancakes etcetera, whereas for other countries these were reported separately.

Our findings are consistent with earlier reports on fat and fatty acid intakes in European 1, 12 and other countries around the world 8, 9. These indicate that in most countries SFA intakes are higher than recommended, while PUFA intakes are often below the optimal intake range. The European TRANSFAIR study reviewed survey data from 14 western European countries conducted between 1980 and 1996. It reported average population intakes ranging from 10–19%E for SFA and from 3–7%E for PUFA 1. The European Prospective Investigation into Cancer and Nutrition study (EPIC) conducted in ten western European countries between 1995 and 2000 reported SFA intakes ranging from 9–16%E and PUFA intakes from 4–8%E across countries 12. In addition, our findings on dietary sources of fat and fatty acids are in agreement with those from the TRANSFAIR study and also with the EPIC study in European populations [1,6]. When our findings are compared to the TRANSFAIR and EPIC data, SFA and PUFA intakes and food patterns in Europe do not seem to have changed much over the past 20–25 years. This is in contrast to TFA intake, which decreased markedly over the past 20–30 years in Europe 17.

High SFA intake is usually accompanied by high total fat intake, whereas PUFA intake seems to be much less dependent of total fat intake 9. This implies that the differences in PUFA intakes between countries are apparently not driven by differences in total fat intakes, but more likely due to differences in local food habits and types and amounts of cooking oils and fats used. For example, high use of sunflower oil in Bulgaria is known to contribute to the relatively high PUFA intake (in particular linoleic acid) in this country 18, whereas high use of margarines and mayonnaise make these foods the largest contributors to PUFA intake in Denmark, Norway, Sweden, Netherlands, Belgium, Ireland, and Poland. At the same time in many Western European countries, the common use of butter and animal fats for cooking importantly contributes to overall SFA intake 6, 19. Thus, there is a large potential for decreasing SFA and increasing PUFA intake by promoting consumption of vegetable oils, margarines, and leaner variants of dairy and meat products.

Changes in intakes of fatty acids can have important effects on the risk of CHD in the population. The available evidence indicates that SFA reduction as such, without considering what nutrient(s) should replace the calories from SFA, is not enough to reduce CHD risk 20. Data from randomized clinical trials 21, prospective cohort studies 22, as well as controlled metabolic studies on blood lipids 23 consistently show that reducing intakes of SFA and trans‐fatty acids (TFA) and replacing these with PUFA reduces the risk of CHD 22, 24, 25. Together, these different types of evidence indicate that substituting 5%E PUFA for SFA will reduce the risk of CHD events by 10% 21. Authoritative expert bodies agree that for prevention of coronary heart disease, dietary SFA can best be replaced by PUFA 2, 3. The data in our review support food‐based guidelines that advice to eat less fatty meats, low‐fat instead of full‐fat dairy, and more vegetable oils. If these are effectively implemented, adherence to these guidelines will reduce SFA and increase PUFA intake in the population. Our review also shows that reliable data on actual intakes of fatty acids in the population are scarce, in particular for countries in Eastern Europe and former USSR. Adequate data on fatty acid intake and their dietary sources are needed to establish and support effective public health policies, including food‐based dietary guidelines.

In conclusion, the available data indicate that the majority of adults living in Europe have higher SFA intakes than the recommended 10%E, and that PUFA are at the low side of the recommended optimal intakes range of 6–11%E. Adhering to food‐based dietary guidelines for prevention of CHD and other chronic diseases in Europe will help to reduce SFA intake and at the same time increase PUFA intake.

5

*Authors would like to thank Unilever colleagues for helping to identify dietary data sources from national public health institutes*.

*Disclosure statement: AE, RKH, and PLZ are employees of Unilever. Unilever markets food products made of vegetable oils, including margarines and dressings*.
